# Surface Functionalization of Ti6Al4V-ELI Alloy with Antimicrobial Peptide Nisin

**DOI:** 10.3390/nano12234332

**Published:** 2022-12-06

**Authors:** Mari Lallukka, Francesca Gamna, Virginia Alessandra Gobbo, Mirko Prato, Ziba Najmi, Andrea Cochis, Lia Rimondini, Sara Ferraris, Silvia Spriano

**Affiliations:** 1 Department of Applied Science and Technology, Politecnico di Torino, Corso Duca degli Abruzzi 24, 10129 Torino, Italy; 2 Faculty of Medicine and Health Technology, Laboratory of Biomaterials and Tissue Engineering, Tampere University, Korkeakoulunkatu 3, 33720 Tampere, Finland; 3 Materials Characterization Facility, Istituto Italiano di Tecnologia, Via Morego 30, 16163 Genova, Italy; 4 Department of Health Sciences, Center for Translational Research on Autoimmune and Allergic Diseases—CAAD, Università Del Piemonte Orientale UPO, Corso Trieste 15/A, 28100 Novara, Italy

**Keywords:** titanium alloy, nisin, surface functionalization, antimicrobial peptide, anti-microfouling

## Abstract

Implant-associated infections are a severe global concern, especially in the case of orthopedic implants intended for long-term or permanent use. The traditional treatment through systemic antibiotic administration is often inefficient due to biofilm formation, and concerns regarding the development of highly resistant bacteria. Therefore, there is an unfulfilled need for antibiotic-free alternatives that could simultaneously support bone regeneration and prevent bacterial infection. This study aimed to perform, optimize, and characterize the surface functionalization of Ti6Al4V-ELI discs by an FDA-approved antimicrobial peptide, nisin, known to hold a broad antibacterial spectrum. Accordingly, nisin bioactivity was also evaluated by in vitro release tests both in physiological and inflammatory pH conditions. Several methods, such as X-ray photoelectron spectroscopy (XPS), and Kelvin Probe atomic force microscopy confirmed the presence of a physisorbed nisin layer on the alloy surface. The functionalization performed at pH 6–7 was found to be especially effective due to the nisin configuration exposing its hydrophobic tail outwards, which is also responsible for its antimicrobial action. In addition, the first evidence of gradual nisin release both in physiological and inflammatory conditions was obtained: the static contact angle becomes half of the starting one after 7 days of soaking on the functionalized sample, while it becomes 0° on the control samples. Finally, the evaluation of the antibacterial performance toward the pathogen *Staphylococcus aureus* after 24 h of inoculation showed the ability of nisin adsorbed at pH 6 to prevent bacterial microfouling into biofilm-like aggregates in comparison with the uncoated specimens: viable bacterial colonies showed a reduction of about 40% with respect to the un-functionalized surface and the formation of (microcolonies (biofilm-like aggregates) is strongly affected.

## 1. Introduction

Metallic materials are one of the main categories of biomaterials employed for musculoskeletal applications. Especially, titanium-based alloys, such as Ti6Al4V typically used for medical implants, and its extra-low interstitial version Ti6Al4V-ELI with improved fracture toughness and corrosion resistance [1], are suitable for medical use. In general, titanium and its alloys are well tolerated by the human body. However, there are still challenges associated with infection, acute and chronic inflammation, osteolysis, implant loosening, and failure [2].

One of the main issues of modern orthopedics is to promote osteointegration and simultaneously prevent infection and biofilm formation. In addition, the increasing threat of antimicrobial resistance has shifted the research focus toward antibiotic-free alternatives [3]. Conventionally, antibiotics are administered systemically to the patient, but since the desired site is often covered by biofilm preventing antibiotic penetration, local delivery of an antibacterial agent or initial prevention of the bacterial adhesion would be more viable options [4]. In addition, local delivery of antibacterial agents could reduce the risk of toxic dosage and resistance building because a lower concentration of antimicrobials would be needed compared to systemic antibiotics [4].

To respond to the medical need for biocompatible and antibacterial orthopedic devices, several strategies to tailor metal implant surfaces are being studied. The surface modification strategy can either indirectly inhibit bacterial adhesion and biofilm formation (antiadhesive or anti-microfouling action) or directly kill adhered bacteria (bactericidal action) [5]. Currently investigated approaches include surface coatings, modification of surface chemistry and topography of the material, and the incorporation of antimicrobial agents into biomaterials [3]. Regarding the non-antibiotic antimicrobial agents, there are several options investigated, such as metal ions and oxides, polymers, enzymes, quorum sensing drugs, bacteriophages, or antimicrobial peptides [3,6,7,8,9,10,11].

Antimicrobial peptides (AMPs) are small, usually cationic peptides with a broad antimicrobial spectrum and immunomodulatory activity against bacteria, viruses, and fungi [12]. One of the most studied and used AMP is nisin, first identified in 1928 in fermented milk cultures. It is better known as a food preservative used in several products including dairy products. Nisin is FDA-approved and implemented worldwide in several food-related applications [13].

**Figure 1:**
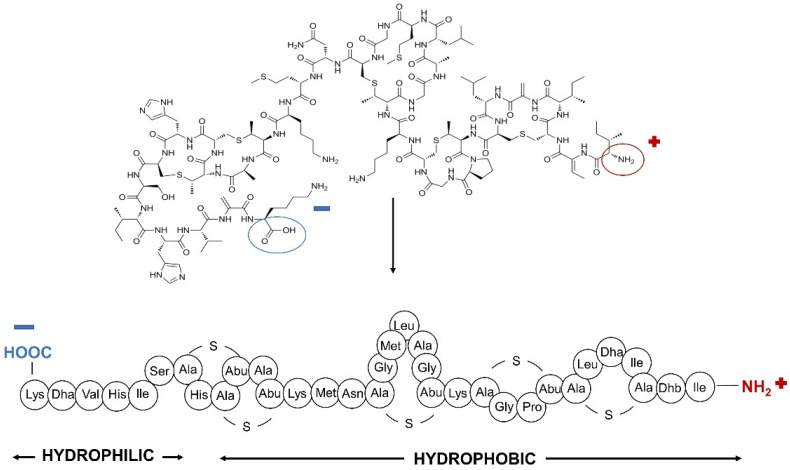
Chemical and primary structures of the nisin polypeptide.

Regarding its structure, nisin (C_143_H_230_N_42_O_37_S_7_) is a pentacyclic post-translationally modified peptide composed of 34 amino acids with a weight of 3354.12 Daltons [14]. It is a class I A lantibiotic bacteriocin produced by *Lactococcus lactis*. Lantibiotics refer to ribosomally synthesized antimicrobial compounds of bacterial origin, which contain unusual amino acids, such as, in the case of nisin, didehydroalanine, didehydroaminobutyric acid, lanthionine, and β-methyl-lanthionine [14]. In addition, the structure of nisin is characterized by several internal disulphide bridges. There are several characterized forms of nisin, nisin A being the most active [15]. Nisin can be considered an amphiphilic molecule since it has both hydrophilic and hydrophobic residues in its structure [15]. The hydrophobic residues of nisin are identified to be responsible for its antimicrobial activity [16,17]. Concerning its stability, nisin is known to be highly stable in acidic pH, in which it can also withstand high temperatures [12]. The chemical structure of nisin is displayed in Figure 1.

Nisin is known to exhibit antimicrobial activity mainly against Gram-positive bacteria. Gram-negative bacteria are problematic because nisin hardly penetrates their outer membrane barrier, and thus reaching its target lipid II in the inner membrane could be challenging. However, nisin is effective also against Gram-negative bacteria when combined with chelating agents, such as ethylenediaminetetraacetic acid (EDTA), citrate monohydrate, trisodium orthophosphate, or with heat treatments, or freezing [16]. The nisin mechanism of action is widely characterized and consists of several steps. Firstly, nisin binds to the anionic phospholipids (lipid II) on the cytoplasmic membrane of the bacteria, inhibiting their cell wall formation. Subsequently, the peptide forms an ion channel or a pore on the membrane, leading to a fatal efflux of intracellular products, such as adenosine triphosphate (ATP) and potassium [18].

Recently, in addition to its exploitation as a preservative in the food industry, the potential of nisin in biomedical applications has gained interest [4]. In the context of infections, the antimicrobial effects of nisin have been reported against mastitis [19], respiratory [20], gastrointestinal [21,22], and skin infections [23]. Moreover, nisin has been found to exhibit selective cytotoxicity toward cancer cells and influence tumor growth [24,25,26]. In addition, besides antimicrobial properties, several studies suggest that nisin can also show immunomodulatory properties mainly by affecting cytokine production [27]. Some evidence of the effect of nisin on innate and adaptive immune cells also has been observed [28]. However, due to various discrepancies between the results and high variability in the experimental models, more studies are therefore required to verify the effect [15].

AMPs can be grafted on the surface with covalent bonds thanks to the amines, carboxylic acids, thiols, and hydroxyls in their inherent structure [29]. Ideally, the peptide should stay stable and in its active form in the coating, without the impact of the environmental conditions on the contact surface that is exposed. Simultaneously, the peptide should be possible to be released from the surface so that it will provide antimicrobial performance [30]. Covalent bonding of nisin has been implemented to stainless steel surfaces by previously applying a chitosan layer to increase the affinity, and the adsorbed peptides were found to decrease bacterial adhesion on the functionalized surface [31]. Immobilization and release of nisin nanoparticles have also been studied from the cracked and uncracked oxide films on stainless steel and titanium [30,32]. In addition to metallic materials, nisin has also been grafted onto glass substrates by using dopamine as a coupling agent [33].

In terms of incorporating AMPs as a coating to biomaterial surfaces, current challenges are related to the possible hydrolysis and denaturation of the coating due to pH and temperature, which could compromise their antimicrobial activity. It is also possible that the activity of AMP decreases because of its functional groups essential for antimicrobial action covalently linking to the surface, rendering the peptide inactive [29].

This work aims to develop a biocompatible and antibacterial surface by nisin functionalization. To the extent of our knowledge, there is no work adsorbing nisin on the surface of Ti64ELI alloy. In this work, the surface functionalization process of the Ti64ELI titanium alloy with antimicrobial peptide nisin has been optimized, without the addition of any toxic linker, varying the process parameters such as the pH value of the nisin solution during the functionalization process. The surface functionalization is evidenced by several methods, followed by release tests both in physiological and inflammatory mimicking conditions assessing the expected mechanism of antibacterial action. Finally, the antibacterial performance of the adsorbed nisin-coating has been assessed towards the *Staphylococcus aureus* (*S. aureus*) strain for 24 h showing promising anti-microfouling activity.

## 2. Materials and Methods

### 2.1. Sample Preparation

Ti6Al4V-ELI (Grade 23, Titanium Consulting and Trading S.r.l., Firenze, Italy) 10 mm diameter 2 mm thickness discs were employed for the experimental study. Discs were polished with SiC abrasive papers up to 4000 grit. The polished discs were then sonicated once for 5 min in acetone, and successively twice for 10 min in ultrapure (Milli-Q) water to remove contaminations eventually present on the sample surfaces. From now on, these samples will be named TI64ELI-MP (mechanically polished).

### 2.2. Functionalization of Sample Surfaces with Nisin 

TI64ELI-MP samples were UV-C-irradiated (UV-C 40 W, 253.7 nm, Philips TUV T8) for one hour before functionalization to activate the surface. Antimicrobial peptide nisin (Nisin Ready Made Solution, 20,000–40,000 IU/mL in 0.02 N HCl, Sigma-Aldrich, Milan, Italy) was used for biological functionalization. A nisin concentration of 1 mg/mL in ultrapure water was used for the study in solutions with three different pH: pH 5, pH 6, and pH 7. The pH was adjusted by buffering the initial solution with 1 M NaOH and 1 M HCl, monitoring it with a pH meter (Edge pH, HANNA Instruments Italia S.r.l., Padova, Italy). An amount of 5 mL of the functionalization solution was used to treat each sample. Samples were left to functionalize for 24 h at room temperature in closed containers. After functionalization, samples were washed twice in ultrapure water and left to dry in the air. From now on, nisin-functionalized samples are referred to as TI64ELI-MP NISIN pH = 3/5/6/7. In addition, control samples (TI64ELI-MP CTRL pH = 5/6/7) were prepared by soaking the TI64ELI-MP samples in 5 mL of ultrapure water, previously pH-adjusted according to the respective pH values.

### 2.3. Physical-Chemical Characterization

#### 2.3.1. Energy-Dispersive X-ray Spectroscopy (EDS)

EDS (JCM-6000 Plus Benchtop SEM, Jeol, equipped with EDS, Tokyo, Japan) was performed on both bare and nisin-functionalized samples to assess the changes in elemental composition using the accelerating voltage of 15 kV.

#### 2.3.2. UV–Vis Spectroscopy and Reflectance

Reflectance measurement was performed for both bare and nisin-functionalized samples by means of a UV–Vis spectrophotometer (UV-2600 Shimadzu, Kyoto, Japan, equipped with ISR-2600Plus integration sphere). The spectrum of 190–750 nm was evaluated in the measurements.

#### 2.3.3. Surface Wettability 

The surface wettability of the samples was evaluated utilizing static contact angle measurement by the sessile drop method (Krüss DSA 100, KRÜSS GmbH, Hamburg, Germany). Ultrapure water was used as a wetting fluid. A drop of water (5 µL) was deposited on the surface with a pipette and the contact angles were measured through the instrument software (DSA-100, Dropshape Analysis, KRÜSS GmbH, Hamburg, Germany).

#### 2.3.4. Zeta Potential

The zeta potential measurements were performed using an electrokinetic analyzer (SurPASS 2, Anton Paar, Graz, Austria), equipped with an adjustable gap cell. The surface zeta potential was measured as a function of pH in a 0.001 M KCl electrolyte solution. Separate couples of TI64ELI discs were used for the two titrations to avoid artifacts due to possible surface reactions occurring during the measurements. First, acidic titration was performed by adding 0.05 M HCl. Secondly, basic titration was performed with 0.05 M NaOH. Four parallel measurements were carried out for each pH point measured.

In addition to solid sample zeta potential measurements, the zeta potential of 1 mg/mL nisin solution was measured using a dynamic light scattering (DLS) particle size and zeta potential analyzer (Nanosizer Nano Z, Malvern Instruments Ltd., Malvern, Worcestershire, UK). Concerning the functionalizing solution, the zeta potential curve was obtained through the measurement of the electrophoretic mobility changing pH step by step from pH 2.5 to 9 by adding HCl 0.05 M or NaOH 0.05 M.

#### 2.3.5. Kelvin Probe Force Microscopy (KPFM)

Nisin 1 mg/mL solution (pH 6) was added on top of the polished sample as a drop and left to functionalize overnight. The goal was to create an interface between bare and functionalized areas on the same sample surface. KPFM (Bruker Innova AFM, Billerica, MA, United States) in tapping mode was used to measure the surface potential of bare and nisin functionalized Ti64ELI, data were elaborated by the software Gwyddion (Gwyddion Version 2.62, Brno, Czech Republic).

#### 2.3.6. X-ray Photoelectron Spectroscopy (XPS)

XPS (Kratos Axis UltraDLD, Kratos Analytical Co., Ltd., Manchester, UK) measurements were performed (survey spectra and high-resolution analyses of elemental regions) on the bare and nisin-functionalized samples to investigate the chemical composition of the outermost layer and, in addition, the presence of characteristic chemical groups for nisin. The hydrocarbon C 1s peak (284.80 eV) was used for the calibration of the binding energy scale.

#### 2.3.7. Release Test 

Release tests were performed following the procedure described in [34]. Shortly, tests were performed both in PBS (pH 7.4, Sigma Aldrich, Milan, Italy) and in hydrogen peroxide (50 mM H_2_O_2_, 30% *w/v*, PanReac Applichem, Monza, Italy; in PBS) with pH adjusted at 4.50 to mimic both physiological and inflammatory conditions, respectively. Each nisin-functionalized sample was soaked in a 5 mL solution for 7 days at 37 °C. The considered time points were 1 and 7 days. Afterward, the samples were washed once with ultrapure water and let to air dry. In addition, the contact angles of the samples were measured after soaking.

### 2.4. Antibacterial Properties Evaluation

#### 2.4.1. Strain Growth Condition 

Bacteria were purchased from the American Type Culture Collection (ATCC, Manassas, VA, USA). Specimens’ antibacterial properties were assayed towards the methicillin/oxacillin (MRSA) resistant *Staphylococcus aureus* strain (Gram-positive, ATCC 43300). Bacteria were cultivated in trypticase soy agar plates (TSA, Sigma-Aldrich, Milan, Italy) and incubated at 37 °C until round single colonies were formed; then, 2–3 colonies were collected and spotted into 15 mL of Luria Bertani broth (LB, Sigma-Aldrich, Milan, Italy) and incubated overnight at 37 °C under agitation (120 rpm). The day after, a fresh broth culture was prepared before the experiments by diluting bacteria into a fresh medium to a final concentration of 1 × 10^3^ bacteria/mL, corresponding to an optical density of 0.00001 at 600 nm wavelength using a spectrophotometer (Spark, from Tecan Trading AG, Mannedorf, Switzerland) [35].

#### 2.4.2. Bacterial Metabolism, Number of Viable Colonies, and Morphology Evaluation

The International Standard ISO 22196 was applied to evaluate specimens’ antibacterial properties [36]. Accordingly, the specimens (Ti64ELI-MP nisin pH6, Ti64ELI-MP nisin pH3, and Ti64ELI-MP, considered as control) were located into a 24-multiwell plate; then, 50 μL of the bacterial suspension was adjusted at a final concentration of 1 × 10^3^ bacteria and it was directly dropped onto the specimens’ surfaces. To confirm the nisin bioactivity at pH = 6, the samples prepared with the pH = 3 nisin stock solution from the manufacturer were also evaluated for their ability in preventing bacterial colonization.

The inoculated specimens were placed in an incubator at 37 °C for 24 h. Afterward, the colorimetric Alamar blue assay (AlamarBlue™, Life Technologies, Milan, Italy) was applied to test viable bacteria metabolic activity by spectrophotometry following the manufacturer’s instructions. Briefly, the ready-to-use Alamar solution at concentration 0.0015% in PBS was added to each well containing the test specimen (1 mL per specimen), and the plate was incubated in the dark for 4 h at 37 °C allowing resazurin dye reduction into fluorescent resorufin upon entering living cells. Then, 100 μL were spotted into a black-bottom 96-well plate to minimize the background signal. The metabolic activity of bacteria was measured via spectrophotometer (λex = 570 nm and λem = 590 nm), and the results were presented as relative fluorescent units (RFU).

Then, to investigate the number of viable bacteria adhered to the samples’ surface the colony forming unit (CFU) count was performed. Briefly, after washing 2 times with PBS to remove non-attached bacterial cells, the samples were submerged into 1 mL of PBS, sonicated, and vortexed for 5 min and 30 s, respectively (three times each). Next, an aliquot of 200 µL of the bacteria suspension was collected and transferred to a new 96 wells plate; here, 6 serials 1:10 dilutions were performed by mixing progressively 20 µL of the bacterial suspension with 180 µL of PBS. Then, 20 µL of each serial dilution were spotted into an LB agar plate and incubated for 24 h until round colonies were visually checked; the final number of CFU was calculated by using the following formula [37]:

CFU = [(number of colonies × dilution factor) (serial dilution)].

Finally, scanning electron microscopy (SEM, JSM-IT500, JEOL, Tokyo, Japan) imaging was used to investigate the bacterial microcolonies formed on the samples’ surfaces; briefly, specimens were dehydrated by the alcohol scale (70–90–100% ethanol, 1 h each), swelled with hexamethyldisilazane, mounted onto stubs with conductive carbon tape and covered with a gold layer. Images were collected at different magnifications (2000× and 5000×) using secondary electrons. Additionally, the presence of single microcolonies or 3D biofilm-like aggregates on the samples’ surfaces as well as their distribution were analyzed through 3D reconstructed images extracted from SEM images using the SMILE VIEW^TM^ map software (JEOL, Tokyo, Japan).

### 2.5. Statistical Analysis of Data

Experiments were performed in triplicate. Results were statistically analyzed using the SPSS software (v.20.0, IBM, New York, NY, USA). Groups were compared by the one-way ANOVA using Tukey’s test as a post hoc analysis. Significant differences were established at *p* < 0.05.

## 3. Results and Discussion

### 3.1. Physical-Chemical Evaluation

First, this research is aimed at verifying the effectiveness of the surface functionalization of the Ti6Al4V-ELI alloy with nisin and comparing different functionalization processes at different pH values (pH = 5/6/7). The pH range of interest has been selected considering that strong acidic or basic solutions could corrode the titanium alloy. The presence of nisin on the functionalized surfaces was evaluated through EDS, XPS (survey quantitative analysis), UV–Vis reflectance, and KPFM.

**Figure 2:**
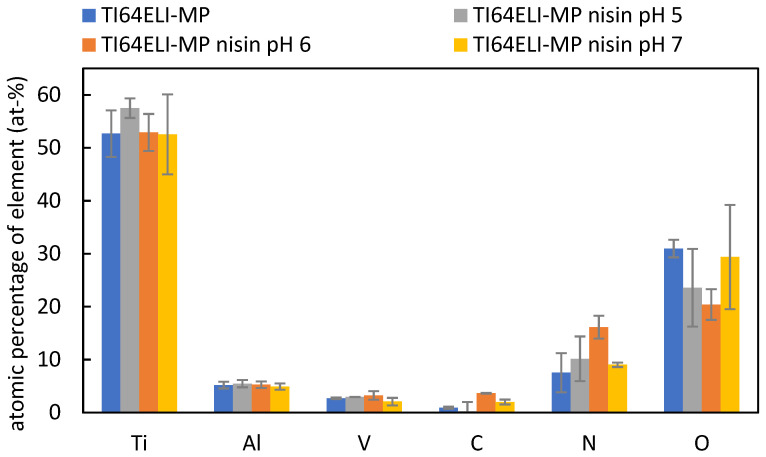
EDS analysis of MP samples, both bare and functionalized surfaces with processes at different pH values (pH = 5/6/7).

The EDS analysis of the bare and nisin-functionalized samples (Figure 2) evidences the presence of all the elements characteristic of the surface of the titanium alloy: Ti, Al, V, and O. A slight increase in nitrogen (N) is noticed comparing the bare and functionalized surfaces, suggesting the presence of the nisin polypeptide on the sample surface; this enhancement is much more significant for the sample functionalized at pH 6, where an increase in carbon (C) is also detectable. Considering the high penetration depth of EDS (higher than 1 micron) and the expected low thickness of the functionalized surface layer, the high standard deviation obtained by this analysis is not surprising. The surface chemical analysis was confirmed through XPS quantitative analysis, which is much more suitable for the chemical characterization of the outermost surface layer thanks to its nanoscale sampling depth, which makes it a more sensitive surface analysis technique [38].

The XPS quantitative analysis of the bare and functionalized surfaces, with processes at different pH values, is reported in Table 1.

The more detailed high-resolution spectra are discussed later in the article. A significant increment of C and N is observed after the functionalization at any pH, with respect to MP as a control. This is coherent with the expected formation of a polypeptide surface layer. Conversely, there is a decrease in Ti, O, and Al on nisin-functionalized surfaces, which is explainable because the titanium oxide layer and metal alloy are covered by the adsorbed nisin layer. S is detected in a too low amount compared to the amount on the MP control sample to be considered significant. No relevant difference among the surfaces functionalized at the different pH is registered by this technique, apart from a slightly higher contribution from the adsorbed polypeptide (C and N) and lower from the elements of the titanium alloy in the case of the sample functionalized at pH 5–6.

**Figure 3:**
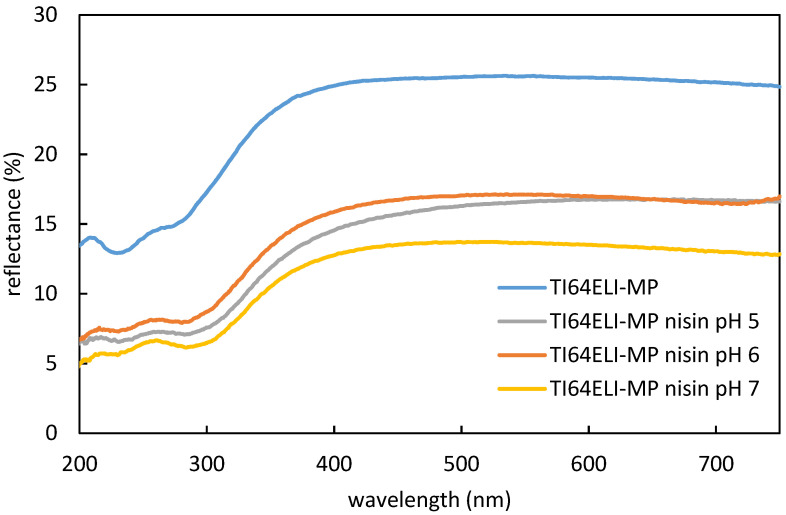
UV–Vis reflectance spectra of the MP samples both bare and surfaces functionalized with processes at different pH values.

The UV–Vis spectra of the bare and functionalized samples have been measured in reflectance (Figure 3).

These data evidence an attenuation, after functionalization, of the surface reflectance along all the measured ranges of wavelength. This agrees with the presence of a surface adsorbed layer able to reduce the high reflectance of the metal surface. No significant difference among the surfaces functionalized at different pH values can be detected by this technique.

**Figure 4:**
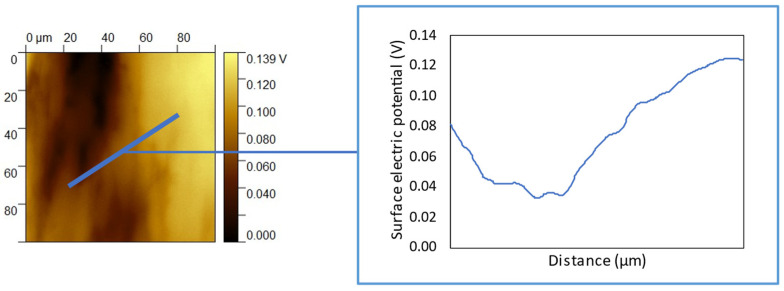
KPFM surface potential as a function of the distance across the interface between a bare (light area) and nisin-functionalized (darker area) region (functionalization at pH 6).

In addition, KPFM has been used for imaging the functionalized surface. A sample functionalized at pH 6 has been chosen for this experiment due to its highest amount of the adsorbed nisin evidenced by EDS and XPS measurements. An internal control surface is needed for this analysis. For this purpose, a sample functionalized only on the half surface and with a sharp border between the functionalized and un-functionalized area has been prepared. When comparing the bare (lighter area) and functionalized (darker) areas of this sample, a clear difference in the surface electrical potential is detected across the interface (Figure 4). This is a further confirmation of the presence of the functionalized surface layer. No evident formation of nisin micrometric aggregates on the surface can be evidenced while the covering seems not homogeneous.

In addition to evidencing the presence of the nisin layer on sample surfaces, further analyses have been performed to characterize the functionalized surfaces: contact angle measurement, zeta potential titration curves, and profile fitting of the XPS data (high-resolution spectra) have been acquired.

**Figure 5:**
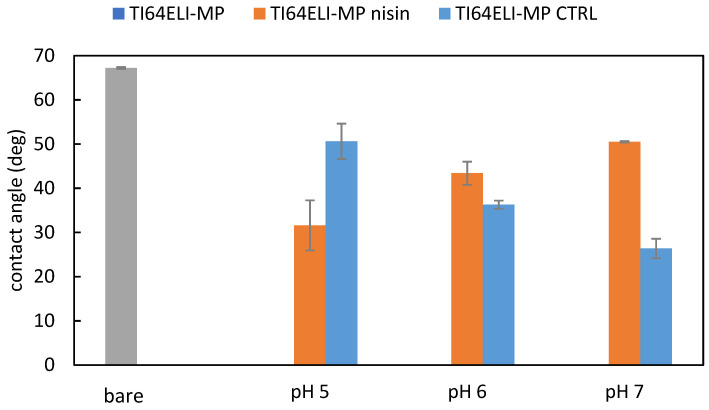
The contact angles measured on the bare MP sample (grey), samples functionalized with processes at different pH values (orange), and control samples soaked in solutions at different pH without nisin (blue).

The modification in wettability due to the functionalization was verified by static contact angle measurements (Figure 5).

It must be considered an eventual contribution due to a modification of the titanium oxide layer during the soaking in solutions at different pH, regardless of the presence of nisin. This effect has been taken into account by considering some control samples soaked, at any pH value, in analogous solutions without nisin (TI64MP-CTRL, blue bars in Figure 4). As displayed in Figure 5, the contact angle of the control samples decreases by increasing the pH of the solution. The opposite phenomenon is observed for the samples functionalized with nisin. At first, these data confirm the presence of nisin on the functionalized samples because of a different wettability than the bare and the control ones. Overall, all the functionalized samples are more hydrophilic than the bare substrate MP, with a maximum difference for the sample functionalized at pH 5. In this case, the presence of nisin induces an increase in wettability even larger than the relative control. A conformation of the adsorbed nisin exposing the hydrophilic moieties outwards is then hypothesized. Contrarily, the presence of nisin on the surface of the samples functionalized at pH 6 and 7 induces a lower wettability with respect to the control samples: a conformation of the adsorbed nisin exposing the hydrophobic moieties outwards can be supposed in these cases. Considering that the hydrophobic tail is the active antibacterial functionality of nisin [39], it can be speculated that this process is much more effective for antimicrobial applications.

**Figure 6:**
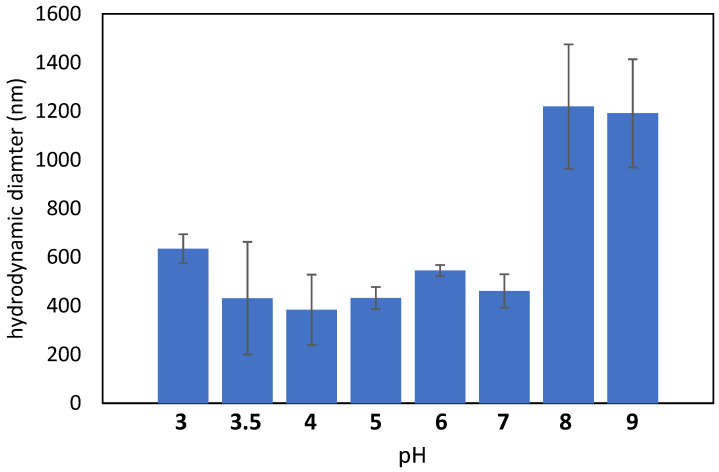
The hydrodynamic diameter of nisin, measured by DLS analysis, in aqueous suspension as a function of pH.

Through DLS, the hydrodynamic diameter and zeta potential of nisin in aqueous suspensions (1 mg/mL) at different pH were measured (Figure 6).

The hydrodynamic diameter is about 400–600 nm at pH between 3 and 7, while it abruptly grows up to 1.2 microns above pH 8 evidencing that the colloidal suspension becomes less soluble and unstable at alkaline pH values. According to these data, the functionalization process cannot be performed at pH equal to or higher than 8, if the formation of agglomerates must be avoided. This is also in agreement with the literature reporting the solubility of nisin to decrease with increasing pH value [40].

**Figure 7:**
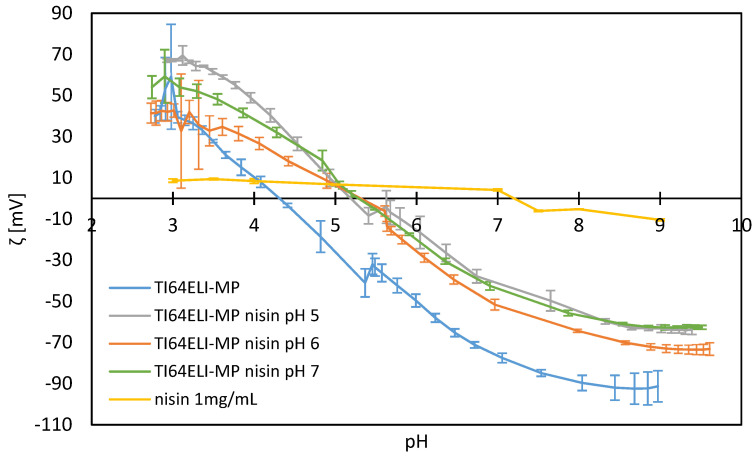
Zeta potential titration curves of the bare MP sample, surfaces functionalized with processes at different pH values, and a solution of nisin.

The zeta potential titration curves of the bare and nisin-functionalized samples are shown in Figure 7, together with the zeta potential titration curve of the nisin solution obtained by DLS.

Different information can be extracted from these curves: the isoelectric point (IEP), the chemical stability of the surface as a function of pH (related to the error bars of the zeta potential), and the expected difference of net charge between the biomolecule and titanium surface during the functionalization process.

The IEP of the bare MP sample is close to 4.5, which is expected for a surface without functional groups with a strong acid–basic behavior. The expected IEP value of nisin is approximately 8.5, based on the literature [40]. It has been experimentally detected here at pH 7.5, the difference with the literature data can be due to the low absolute value of the measured zeta potential and high sensitivity of IEP by changing the electrolytic solution. In agreement with the detected IEP, an increase in the hydrodynamic radius has also been registered above this value of pH, as previously reported.

The IEP of the nisin-functionalized samples can be seen to shift to more basic pH values than the MP sample, in the direction of the nisin IEP, indicating a successful and similar functionalization with nisin on all surfaces. The IEP of the functionalized samples is different from the nisin one, suggesting that there is not a covering coating on the surface, but a layer of adsorbed molecules that allow the exposition of the substrate to the solution.

The error bars of all the functionalized surfaces are the lowest in the pH ranging from 7.5 down to 4.5, which is the region of pH expected to occur in physiological and inflammatory conditions, respectively. This means that the adsorbed nisin is expected to be chemically stable on the surface even if the surrounding chemical environment changes in pH after implantation.

Lastly, it is crucial to highlight the difference in the sign of the net charge detected on the MP sample and the one characterizing nisin solution at the pH values used for functionalization (pH 5, 6, 7). Physisorption with an electrostatic attraction between the negatively charged metal surface and the overall positively charged polypeptide can be expected during the functionalization processes. Considering that nisin at pH 7 is close to its IEP (almost no net charge on the biomolecule at pH 7) and, on the other side, that the absolute value of the zeta potential of the bare TI64ELI-MP is the lowest at pH 5 (minimum surface charge of the substrate within the explored range of pH for functionalization), the highest electrostatic interaction between the substrate and the physisorbed biomolecule can be expected to occur at pH 6.

**Figure 8:**
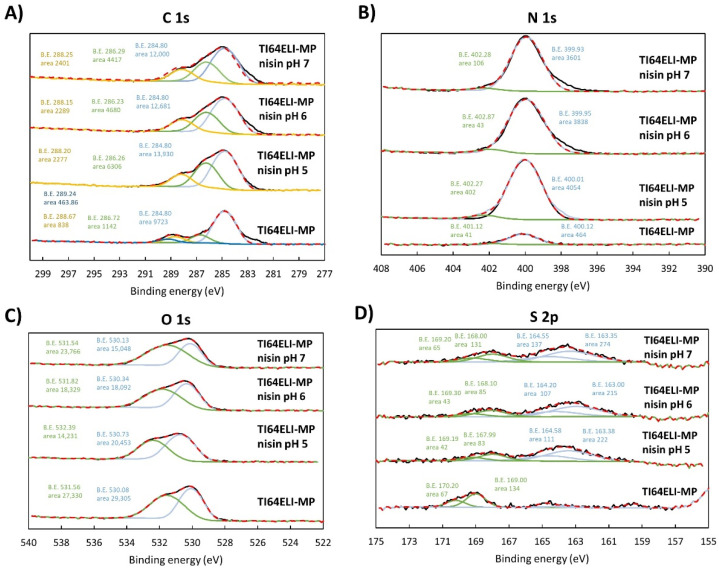
High-resolution spectra of (**A**) C 1s, (**B**) N 1s, (**C**) O 1s, and (**D**) S 2p regions of the bare MP sample and surfaces functionalized with processes at different pH values. Black line (-): spectrum line, red dashed line (- - -): composite line.

The high-resolution spectra of the C 1s, N 1s, O 1s, and S 2p region of the bare MP sample and surfaces functionalized with processes at different pH values are reported in Figure 8.

Concerning the C peak (Figure 8A), the peaks around 286 eV (C-O) and 285 eV (C-C) are higher on all the functionalized samples compared to the bare sample. An additional peak around 289 eV is evident on the bare sample, and it could relate to -COOH groups, while the one around 288 eV might be due to deprotonated ones.

The N peak (Figure 8B) is significantly higher on the functionalized samples, as already noticed, according to the presence of an adsorbed layer of the polypeptide. The peak coming from the charged/protonated amino groups (NH_3_^+^) at 401.9 ± 0.2 eV is always very low with respect to the peak of the neutral aminic groups (NH_2_) at 400.1 ± 0.2 eV, as evidenced by the profile fitting. This can be explained by hypothesizing a physisorption mechanism for the functionalization, which would be coherent with the absence of any functional group with a net charge or high chemical reactivity on the substrate.

The presence of the adsorbed layer on the functionalized samples as a non-covering layer is confirmed by the profile fitting of the oxygen region (Figure 8C). The peak due to the Ti-O bond (~530 eV) of the titanium oxide layer is always observable. The peak at around 531.5 eV can be attributed to the OH -functional groups exposed by MP. This analysis consequently shows that the MP substrate is not completely lacking in functional groups. Considering also the information obtained from the zeta potential titration curve, it can be concluded that the MP substrate has hydroxyl groups, but they do not have a strongly acidic or basic behavior and are not easily protonated or deprotonated in contact with liquid media. Consequently, they are not available for a chemisorption mechanism. A shift of this peak due to the presence of the peptide bond is observed in the functionalized samples. This is more evident in the case of the sample functionalized at pH 5, in agreement with the higher C and N amount detected on this sample in the survey chemical analysis (Table 1).

Concerning the profile fitting of the S region (Figure 8D), a contribution of the thioether bridges -C-S-C- coming from the nisin backbone is observable on the functionalized samples. Regarding S high-resolution XPS data, each chemical state corresponds to a double of peaks, in the case of C-S-C (163 eV and 164 eV) [41]. The other detected S state, with the peak doublet approximately at 168 eV and 169 eV, could be due to the contribution of sulphonate groups (C-SO_3_-H). However, as shown in the XPS survey scan (Table 1), no sound conclusion about the S contribution can be made due to the low detected amount similar to control samples without nisin.

**Figure 9:**
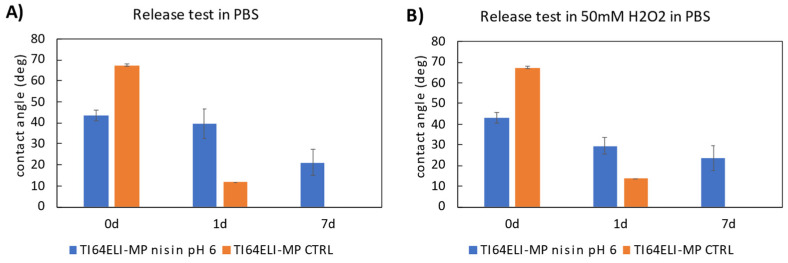
(**A**) The contact angles measured from non-soaked (0 d) and PBS- soaked (1 d, 7 d) samples. (**B**) The contact angles measured from non-soaked (0 d) and H_2_O_2_- soaked (1 d, 7 d) samples. Functionalization has been performed at pH 6.

Once characterized by the functionalized surfaces, two release tests have been performed to investigate the expected mechanism of action of the functionalized surface once in contact with a liquid environment chemically close to the physiological one. The samples functionalized at pH 6 have been used as an example, based on their expected high nisin content and the highest electrostatic difference between the substrate and the polypeptide, as discussed earlier. A control MP sample (without any functionalization layer) has been soaked in the same solution. The solution used for the first release test is PBS to mimic the physiological chemical environment. A second solution has been exploited to mimic an inflammatory chemical environment. It was obtained by adding hydrogen peroxide to PBS and reducing pH down to 4.5 [42]. The eventual change in the surfaces after the release tests has been monitored by the static contact angle measurements. The contact angle results of the release test are reported in Figure 9.

A decrement in the contact angle is observed over the soaking time in both solutions. However, the contact angles of the functionalized and control samples are notably different, both after 1 and 7 days of soaking: this could mean that nisin is only partially released even after a week of soaking. According to these data, a double mechanism of action can be expected from the functionalized surface in a biological environment, through both release in the surrounding liquids (for a time longer than 1 day) and direct contact with cells and tissues (for a time shorter than 1 day). These data agree with the titration curves of the functionalized samples, where the surfaces show a stable zeta potential even changing the pH. Finally, a long-lasting effect can be expected by the functionalized surfaces, even longer than one week, which is not common concerning other functionalized surfaces [43].

To summarize, the presence of a physisorbed layer of nisin on the surface of functionalized samples was confirmed by several methods, such as EDS, XPS, and KPFM. In addition, the functionalization performed at pH 6 was found to favor the nisin configuration exposing its hydrophobic tail outwards, which is also known to be responsible for its antimicrobial action and to maximize the physisorption through electrostatic attraction. Finally, as confirmed by the in vitro nisin release tests both in physiological and inflammatory conditions, evidence of gradual nisin release was obtained.

### 3.2. Antibacterial Properties Evaluation

**Figure 10:**
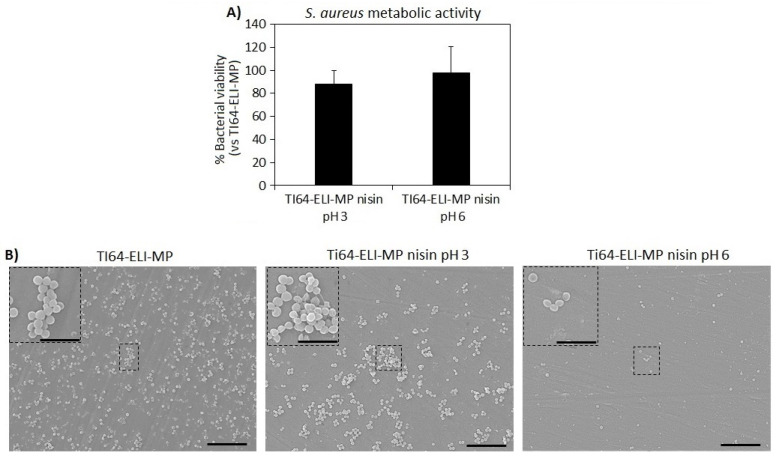
Antibacterial activity evaluation of Ti64ELI-MP functionalized with nisin at pH 3 and pH 6 after 24 h; (**A**) metabolic activity of bacterial cells normalized towards bare substrate Ti64ELI-MP; (**B**) SEM images at two magnifications: 2000× (scale bare = 10 µm) and 5000× (scale bar = 5 µm).

Finally, the antibacterial evaluation of the nisin-functionalized surfaces concerning Ti64ELI-MP (control specimen) was performed towards *Staphylococcus aureus* (*S. aureus*) which was selected as a reference strain due to its common involvement in bone infections. Moreover, specimens obtained by functionalization in the diluted stock solution of nisin at the original pH (pH = 3) were here exploited as a reference due to the known nisin sensibility to pH variation. Therefore, the Ti64ELI-MP nisin pH 6 was compared both to the bulk material and to the Ti64ELI-MP nisin pH 3 as well. The results of the colorimetric metabolic assay Alamar blue and SEM images are reported in Figure 10A,B, respectively, whereas Table 2 shows the viable bacterial colonies number (at serial dilution 10^5^) from the CFU assay.

In general, according to the metabolic activity assay, the samples functionalized with nisin at both pH 3 and pH 6 do not seem to show any statistically significant differences concerning bare Ti64ELI-MP substrates (Figure 10A, *p* > 0.05). However, viable bacterial colonies counting at serial dilution 10^5^ (Table 2) showed a reduction of about 40% and 28% in colonies number for Ti64ELI-MP nisin pH 6 in comparison to bare Ti64ELI-MP and Ti64ELI-MP nisin pH 3 samples’ surfaces, respectively; this reduction is clearly noticed in SEM images collected from sample surfaces after 24 h of direct infection with bacteria (Figure 10B). In fact, mostly single-round colonies were detected on the nisin-functionalized surfaces at pH 6 (Ti64ELI-MP nisin pH 6) whereas on the control (Ti64ELI-MP) and nisin-functionalized surfaces at pH 3 (Ti64ELI-MP nisin pH 3) the formation of many 3D microcolonies (biofilm-like aggregates) was observed. Therefore, a promising anti-microfouling activity preventing the formation and maturation of microcolonies (biofilm-like aggregates) seems to be obtained by the functionalization with nisin, and the efficacy of the protocol at pH 6 is confirmed.

**Figure 11:**
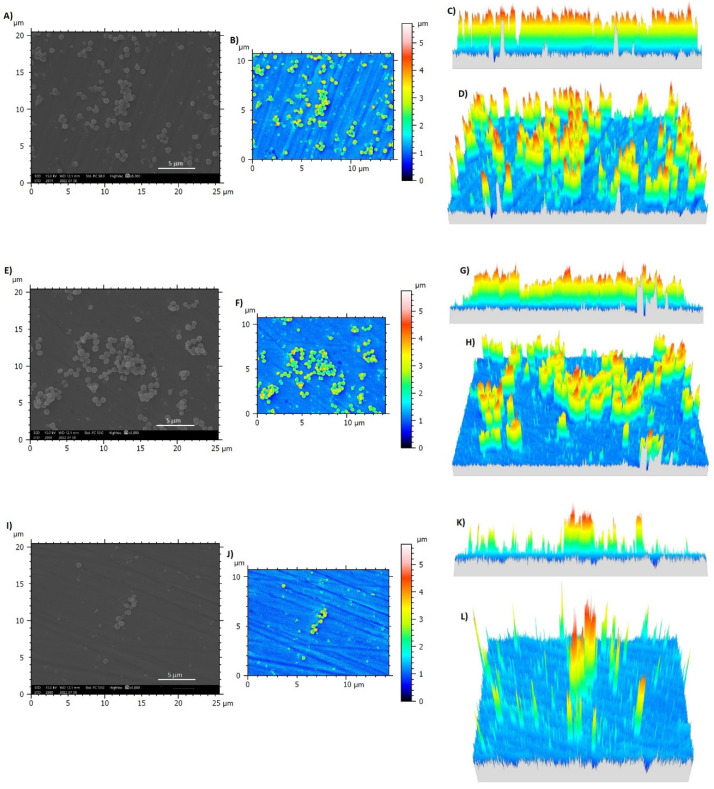
Three-dimensional reconstructed images of bacterial microcolonies on samples’ surfaces extracted from SEM images with SMILE VIEW^TM^ software. Ti64ELI-MP: (**A**) SEM image at magnification 2000× (scale bar= 5 µm); (**B**) reconstructed 3D image extracted from (**A**); (**C**) cross-section image of the bacterial microcolonies on the sample surface; (**D**) whole view of bacterial microcolonies from the selected section; Ti64ELI-MP nisin pH 3: (**E**) SEM image at magnification 2000× (scale bar= 5 µm); (**F**) reconstructed 3D image extracted from (**E**); (**G**) cross-section of the bacterial microcolonies on the sample surface; (**H**) whole view of bacterial microcolonies from the selected section; Ti64ELI-MP nisin pH 6: (**I**) SEM image at magnification 2000× (scale bar= 5 µm); (**J**) reconstructed 3D image extracted from (**I**); (**K**) cross-section of the bacterial microcolonies on the sample surface; (**L**) whole view of bacterial microcolonies from the selected section. Bars indicate the height of microcolonies (µm).

To investigate this activity in detail, 3D reconstructed images were prepared from SEM images at magnification 2000× and shown in Figure 11. As reported in the cross-section of 3D reconstructed images of Ti64ELI-MP (Figure 11C,D, extracted from SEM image of Ti64ELI-MP shown in Figure 11A) and Ti64ELI-MP nisin pH 3 (Figure 11G,H; extracted from SEM image of Ti64ELI-MP nisin pH 3 shown in Figure 11E), almost all *S. aureus* formed 3D microcolonies aggregates on the samples’ surfaces; in fact, Figure 11B,F show that the size of such microcolonies range between 4–5 µm for both Ti64ELI-MP and Ti64ELI-MP nisin pH 3 samples in comparison to the size of *S. aureus* of about 1–1.5 µm indicating that these 3D microcolonies are made up of about 3–4 layers of bacterial cells. The calculation of occupied surfaces with biofilm-like aggregates revealed 19.1% and 16.39% on the Ti64ELI-MP and Ti64ELI-MP nisin pH 3 were colonized by *S. aureus*, respectively (these data were extracted from the field of view of Figure 10B,F as representatives of whole samples’ surfaces). Surface analysis of 3D reconstructed images of Ti64ELI-MP nisin pH 6 (Figure 11K,L; extracted from SEM image of Ti64ELI-MP nisin pH 6 shown in Figure 11I) indicates that few bacterial colonies were able to form aggregates of more than 4 µm and most *S. aureus* remained as single cells (Figure 11J,K,L); additionally, only 6.8% of the sample surface was occupied by bacterial microcolonies (these data were extracted from the field of view of Figure 11J as a representative of whole samples’ surfaces) in comparison to 19.1% for Ti64ELI-MP and 16.39% for Ti64ELI-MP nisin pH 3. From the results obtained from the metabolic activity, viable bacterial colonies count (at serial dilution 10^5^), SEM, and 3D reconstructed images analysis it can be concluded that Ti64ELI-MP functionalized with nisin at pH 6 have no bactericidal activity; however, it successfully prevented the bacterial aggregation into 3D biofilm-like aggregates thus reporting a promising antifouling activity. Similar results were found in another study where the synergistic antifouling properties of nisin and dopamine adsorbed to microstructured glasses with different sizes of grooves were reported against *Bacillus* sp. with respect to the control sample after 16 h [33]. Additionally, this similar effect was previously obtained by the authors introducing a controlled nano-topography onto Ti alloys by electron beam technology that prevented aggregates formation through a physical hindrance [44]; here, the nisin seems to play a similar role but via biochemical induction acting as an anti-aggregation signal for adhered bacteria. Few comparable works can be found in the literature, but the presence of nisin was previously shown by Blackman et al. [45] to reduce the surface contamination from bacterial aggregates in combination with a specific patterning as well as Kim et al. [46] demonstrated that the presence of nisin conferred outstanding fouling resistance to ultrafiltration PDMA membranes when infected.

One possible explanation for the observed ability of the nisin layer to prevent bacterial aggregates is the impact on biofilm maturation. In general, the formation of biofilm is known to consist of several steps including bacteria adhesion, irreversible attachment, biofilm maturation, bacteria dispersal, and bacterial migration. In addition, materials can behave as either anti-microfouling with bacteriostatic action, or as bactericides, acting with different mechanisms and on different stages of biofilm formation [47]. It can be speculated that the nisin-functionalized surface influences the maturation of the biofilm, therefore preventing the aggregation of bacteria. In the literature, nisin is also found to affect the composition and structure of the biofilm. Andre et al. evidenced the presence of nisin to result in a reduction of *S. aureus* biofilm polysaccharides and extracellular DNA, which can be associated with disrupted or decreased biofilm formation [48].

However, it seems evident that even though adsorbed nisin at pH 6 could prevent the formation of bacterial aggregates on the surface to some extent, the well-known antibacterial potential of nisin was not here fully maintained after surface functionalization. Similar results were found in another study where the antibacterial properties of nisin adsorbed to stainless steel against *Listeria monocytogenes* were similar with respect to control after 24 h [49]. The results obtained from the functionalization by using the nisin solutions at pH 3 and pH 6 seem to exclude the hypothesis that the low antibacterial activity is due to the molecule’s sensitivity to pH variation: the functionalization at pH6 is revealed to be more effective in surface grafting and against microfouling and biofilm maturation. The contact-killing activity of nisin is mainly due to its binding with the bacterial membrane causing irreversible deadly pores [50]. So, further studies are still needed to improve the antibacterial efficacy of nisin, as well as other functionalization mechanisms, which could also be investigated to improve either the exposure of nisin’s antibacterial groups or the release of the peptide to the solution.

## 4. Conclusions

The goal of this study was to characterize and optimize the surface functionalization of Ti6Al4V-ELI discs by the antimicrobial peptide nisin. The presence of nisin as a physisorbed layer on the surface was confirmed by different methods. It exposes the hydrophobic and anti-bacterial functionality toward the external environment when functionalization is performed at pH 6–7. In addition, the first evidence of gradual nisin release both in physiological and inflammatory conditions and as well as an anti-microfouling activity against bacteria, and the possible effect on biofilm maturation was preliminarily obtained.

## Figures and Tables

**Table 1:** XPS quantitative analysis of the bare MP sample and surfaces functionalized with processes at different pH values. Error for all the values is indicated at 0.1 at-%.

Elements (Atomic Percentage, at-%)	TI64ELI-MP	NISIN pH 5	NISIN pH 6	NISIN pH 7
C	35.9	64.3	60.2	56.2
O	43.3	24.4	25.3	28.4
N	0.5	4.7	4.5	4.2
Ti	11.1	4.3	5.4	6.0
Al	3.4	0.6	2.0	1.6
S	0.3	0.2	0.2	0.3
Others	5.5	1.4	2.6	3.2

**Table 2:** Viable bacterial colonies number (CFU count, means ±dev.st) after 24 h specimens’ direct infection.

Specimen	Viable Colonies Count (CFU, ×10^5^)
TI64ELI-MP	11 (±1)
TI64ELI-MP nisin pH 3	9 (±0.5)
TI64ELI-MP nisin pH 6	6.5 (±0.5)

## Data Availability

Data can be available upon request from the authors.
